# Antifungal Properties of *Sargassum cinereum* and *Padina boergesenii* Extracts Against Fungi Associated with Strawberry Fruits Concerning Mycotoxin Production

**DOI:** 10.3390/plants13223115

**Published:** 2024-11-05

**Authors:** Amany A. El-Shahir, Nurah M. Alzamel, Amani Omar Abuzaid, Naglaa Loutfy, Eman A. Alwaleed

**Affiliations:** 1 Department of Botany and Microbiology, Faculty of Science, South Valley University, Qena 83523, Egypt; amanyattanew@yahoo.com (A.A.E.-S.); naglaa.hasssn@sci.svu.edu.eg (N.L.); eme_biologist@sci.svu.edu.eg (E.A.A.); 2 Department of Biology, College of Sciences and Humanities, Shaqra University, Shaqra 11961, Saudi Arabia; 3 Biology Department, Faculty of Science, King Abdulaziz University, P.O. Box 80200, Jeddah 21589, Saudi Arabia; aabuzaid@kau.edu.sa

**Keywords:** strawberry, antifungal, mycotoxin, seaweeds, *Sargassum*, *Padina*, *Aspergillus*, *Botrytis*, *Mucor*

## Abstract

Strawberries are susceptible to decay and destruction while being harvested and stored. This study had the following objectives: (1) the documentation of fungi and mycotoxin production associated with infected strawberry fruits; (2) the evaluation of the primary phytochemicals of *Sargassum cinereum* and *Padina boergesenii* by gas chromatography–mass spectrometry (GC–MS) and Fourier transform infrared (FT-IR) analysis to identify the active chemical composition of the seaweed extracts; and (3) the assessment of the antifungal activity of five extracts from brown seaweeds both in vitro and in vivo against fungal infections on fresh fruit under post-harvest conditions. The most common fungi were *Aspergillus niger* 14.36%, *Botrytis cinerea* 38.29%, and *Mucor irregularis* 16.88%. *Padina boergesenii* acetone extract had the highest in vitro antifungal activity. The methanol extracts of both *S. cinereum* and *P. boergesenii* were effective against the pathogenicity and aggressiveness (in vivo) on post-harvest strawberry fruits. *B. cinerea* could produce botrydial and dihydrobotrydial toxins with concentrations of 8.14 µg/mL and 4.26 µg/mL, respectively. *A. niger* could produce ochratoxin A with a concentration of 10.05 µg/mL. The present study demonstrates that the extracts of macroalgae *S. cinereum* and *P. boergesenii* contain secondary metabolites and antioxidants, indicating their potential utilization in antifungal applications.

## 1. Introduction

One of the most popular berries consumed worldwide, the strawberry (*Fragaria* × *ananassa* Duch.; family: *Rosaceae*) is a significant and essential fruit because of its distinct flavor and numerous nutritional and health advantages [1]. Nevertheless, during harvesting and storage, strawberries may be subjected to mechanical damage and decay, which increases their vulnerability to a variety of phytopathogens, such as bacteria, fungi, and viruses [2,3]. The global plant microbiome may also be influenced by continental location and agricultural techniques. Additionally, the fruits’ microbiome affects how long they last on the shelf [4]. Based on early research, the most common diseases that damage strawberries are fungi from the genera *Botrytis*, *Penicillium*, *Phytophthora*, *Verticillium*, *Alternaria*, *Cladosporium*, *Aureobasidium*, *Cryptococcus*, and *Rhizopus* [5]. According to the previous results [6], the two most prevalent genera that affected strawberry fruits were *Botrytis* and *Cladosporium*. Grey mold, or *Botrytis cinerea,* is considered one of the main reasons for post-harvest losses [7,8]. Strawberry yield losses due to fungal infections, particularly *B. cinerea*, can reach 50% when the right conditions are met, such as high humidity, moderate temperature, and overripe fruit [9].

Since some of the mycotoxins produced by fungi are toxic to people and animals, they are significant in determining fungal pathogenicity and food safety. The most prevalent mycotoxins include ochratoxins, aflatoxins, and *Alternaria* toxins. Both humans and animals are exposed to these toxins, which have genotoxic, mutagenic, carcinogenic, DNA strand breakage, gene mutation, and cytotoxic effects, and suppress enzyme activity [10,11,12].

Various strategies are needed to manage fungal infection in strawberries after harvest. The most widely used procedure is the application of fungicides. However, this is expensive, pollutes the environment, and may eventually lead to the emergence of resistant infections [13,14,15]. Recently, fungicide used during the post-harvest phase has been completely prohibited in several European nations [16].

Therefore, efforts have been made to identify alternate pathogen control methods that are safe, harmless to the environment, and pathogen-specific. Seaweed use was assessed to determine its potential for environmentally friendly fungal disease management [12,17,18].

Bioactive natural substances found in marine settings are absent from terrestrial natural goods. Many naturally occurring antimicrobial compounds with biological and pharmacological effects can be found in marine species such as macroalgae [19,20].

Agar, algin, and carrageenan, raw materials utilized in numerous industrial sectors, are among the various resources that seaweeds offer. Algae can contain varying levels of bioactive chemicals, including secondary metabolites, that may have antiviral, antibacterial, and antifungal effects, depending on their phylum, growth stage, and environmental circumstances [21,22]. According to Reshma et al. [23] the majority of synthetic fungicides have the potential to be hazardous to the environment and to leave detrimental residues in crops and soil. Since the 2000s, there has been a greater focus on the use of seaweed extract to prevent post-harvest losses in fruit. According to Kamel [24], fruit quality can be enhanced and preserved longer when used alone. The study examined the effects of a commercial seaweed extract on fruit quality during cold storage.

The objectives of this study were to isolate and identify fungi and mycotoxin production associated with strawberry fruits and to assess the antifungal activity of solvent extracts of *Sargassum cinereum* and *Padina boergesenii* both in vitro and in vivo against fungal infections on fresh fruit under post-harvest conditions. Antifungal chemicals were determined utilizing GC–MS, and Fourier transform infrared (FT-IR) analysis. The crude extract was analyzed for bioactive chemicals or phenolic compounds to gather preliminary data on their potential for antifungal activity.

## 2. Results

### 2.1. Primary Phytochemicals of S. cinereum and P. boergesenii

The ethyl acetic extract of *S. cinereum* and *P. boergesenii* showed the presence of four compounds, including terpenoids, flavonoids, polyphenols, and quinones, in *S. cinereum*. and *P. boergesenii,* polyphenols were present in abundance. Quinones were present in moderate amounts in *S. cinereum*. The others, such as terpenoids and flavonoids, were present in minor amounts in *S. cinereum*. In *P. boergesenii,* flavonoids and quinones were at low levels (Table 1).

The acetonic extract of *S. cinereum* and *P. boergesenii* showed the presence of four compounds; quinones were present at high levels in *S. cinereum* and at low levels in *P. boergesenii*. Polyphenols were present in moderate amounts in both seaweed extracts. The other compounds, such as terpenoids and tannins, were present at low levels in both seaweed extracts (Table 1 and Table 2).

Similarly, the methanolic extract of *S. cinereum* contained five compounds, including alkaloids, terpenoids, flavonoids, polyphenols, and quinones, and the same compound in *P. boergesenii* except with the terpenoid compound replaced by tannins. Polyphenols and quinones were highly present in *S. cinereum* and *P. boergesenii* in the case of methanolic extract. The other compounds were poorly present in both seaweed extracts (Table 1 and Table 2). In the present study, alkaloids were highly present in the ethyl acetate extract of *S. cinereum*.

### 2.2. FTIR Analysis

**Figure 1:**
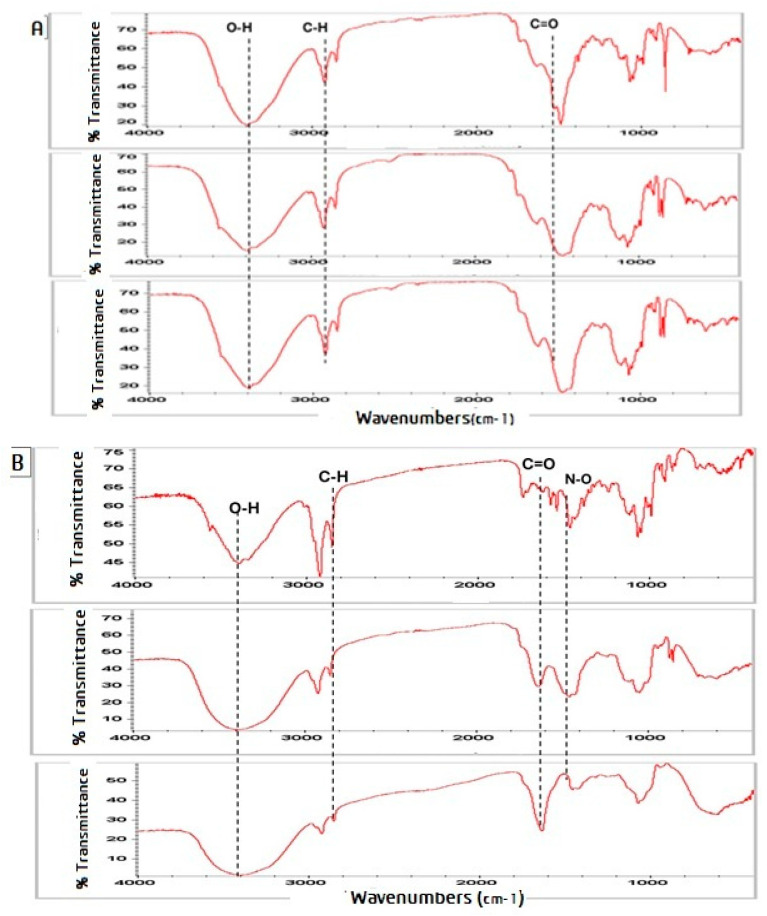
FTIR analysis of acetone, ethyl acetate, and methanol extract of (**A**) *P. boergesenii* and (**B**) *S. cinereum*.

*S. cinereum* and *P. boergesenii* extracts in three different solvents (methanol, acetone, and ethyl acetate) revealed far-off peaks that indicated different functional groups in the 4000–400 cm^−1^ range. The results of the FTIR analysis, which confirmed the presence of O–H, N–H, C–H, C=O, C–C, C–N, and S=O bonds at different extracts, validated the presence of phenols, carboxylic acids, alkoxy, aromatic, alkene, amides/amines, and sulfonate compounds (Table 3 and Figure 1). A range of peaks were visible in the seaweed extracts’ single bond area (2500–4000 cm^−1^). H-bonded alcohols and phenols’ O–H stretch is responsible for the peaks at 3493.5, 3492.6, 3467.1, 3426.5, 3396.2, and 3397.5 cm^−1^. Regarding *S. cinereum*, the major alcohol’s C–O stretch is the cause of the strong peak at 1026.3 and 1067.5 cm^−1^. The phytochemical evaluation of several crude extracts indicated a variety of bioactive compounds with variable concentration levels.

### 2.3. The GC–MS Chromatogram of the Acetone Extracts of Seaweeds

**Figure 2:**
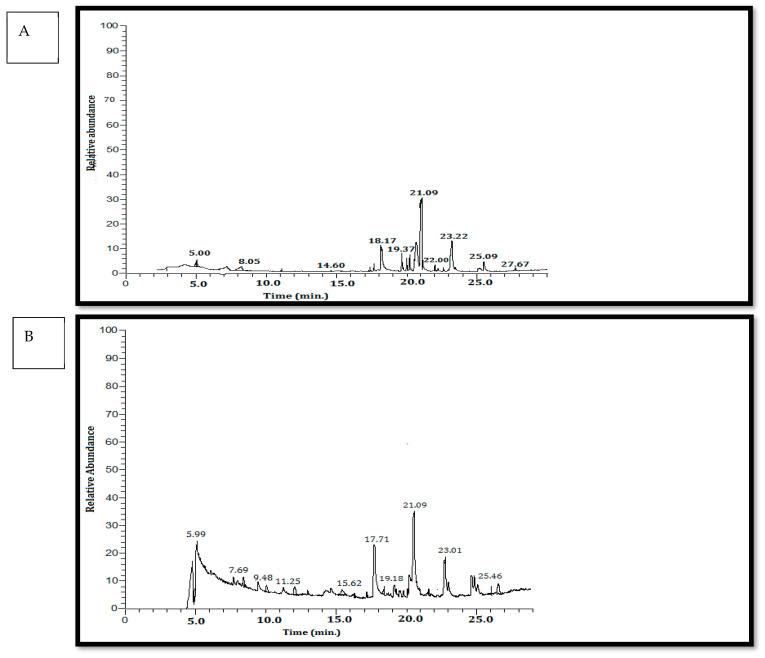
The GC–MS chromatogram of the acetone extracts of (**A**) *S. cinereum* and (**B**) *P. boergesenii*.

The GC–MS chromatogram of the acetone extracts of *Sargassum cinereum* and *Padina boergesenii* harvested from the Hurghada Coastal area exhibited 15 peaks (Figure 2) of which 13 peaks were identified. The chemical compositions of the acetone extract are shown in (Table 3).

The most abundant six constituents were heptadecane (RT = 17.68 min), methyl tetradecanoate (RT = 19.64 min), hexadecanoic acid, methyl ester (RT = 21.09 min), 11-eicosenoic acid, methyl ester (RT = 25.49 min), lucenin (RT = 27.67 min), and docosanoic acid, and methyl ester (CAS) (RT = 29.95 min). Figure 2 identified and quantified the chemical constituents of seaweed and indicated that the algae contains monoterpenes, triterpenes, acyclic alkanes, alkenes, aromatic compounds, and organo-sulfur compounds.

### 2.4. Mycobiota Analysis

**Figure 3:**
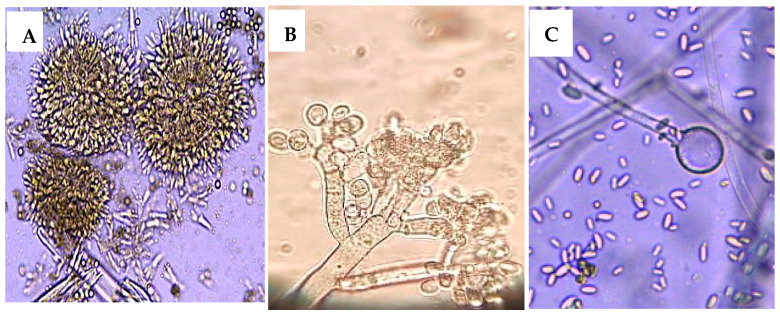
(**A**) *Aspergillus niger* voucher MIC:123 (OR518653), (**B**) *Botrytis cinerea* culture SVU:23 (OR594161), and (**C**) *Mucor irregularis* cultivar (OR578709).

Twelve species of 10 genera were collected from thirty infected strawberry fruits by baiting method on PDA medium at 28 °C. The most common fungi were *Aspergillus niger* (14.36%), *Botrytis cinerea* (38.29%), and *Mucor irregularis* (16.88%) (Table 4 and Figure 3)

### 2.5. Molecular Identification

**Figure 4:**
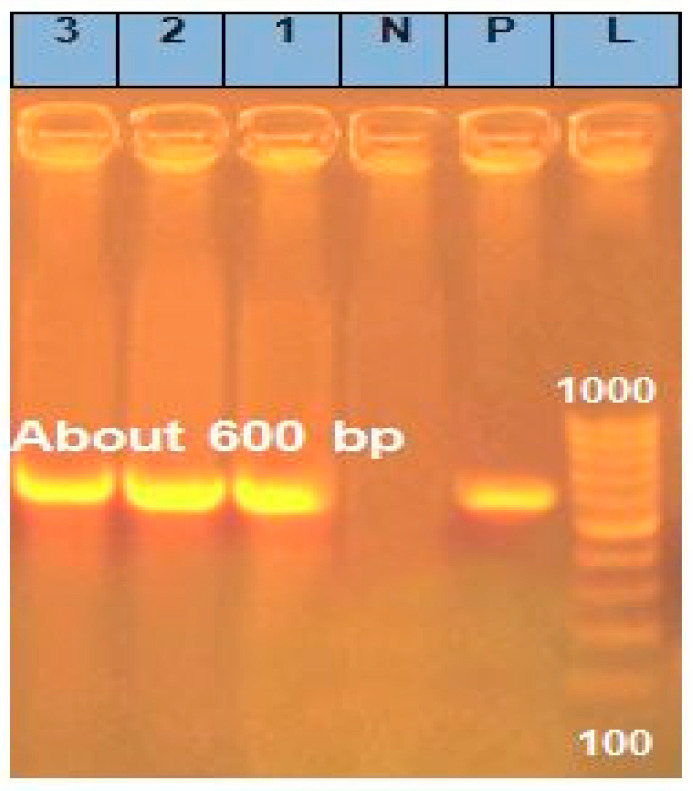
Agarose gel electrophoresis of PCR amplification of DNA products (~600 bp) using ITS1/ITS4 primer pair. L—ladder (100–1000 bp); P—positive control consisting of a segment of DNA of known size (the same size as the target amplicon, showing that the primers have attached to the DNA strand); N—negative control consisting of a sample without DNA, but containing all essential components of the amplification reaction to show if contamination of the PCR experiment with foreign DNA has occurred; and 1—*Aspergillus niger*, 2—*Botrytis cinerea*, and 3—*Mucor irregularis*.

ITS1 and ITS4 rDNA gene sequencing was used to molecularly identify *Aspergillus niger*, *Botrytis cinerea*, and *Mucor irregularis*, the three most common species detected in strawberry fruits. ITS1 (forward) and ITS4 (reverse), two fungal-specific universal primer pairs, were successfully used to amplify the ITS region from the DNA of each *Aspergillus niger*, *Botrytis cinerea*, and *Mucor irregularis* sample. According to electrophoresis, the fragment sizes were around 600 bp (Figure 4). The ITS gene sequencing results for *Aspergillus niger*, *Botrytis cinerea*, and *Mucor irregularis* were 574, 470, and 810 bp, respectively. A BLAST examination of the ITS rDNA sequences verified the morphological identification. The NCBI GenBank database shows that several species of *Aspergillus niger*, *Botrytis cinerea*, and *Mucor irregularis* have the closest match (98–99% similarity). The ITS rDNA sequences of the three species have been added to the NCBI GenBank database under the following names: *Mucor irregularis* cultivar (OR578709), *Aspergillus niger* voucher MIC:123 (OR518653), and *Botrytis cinerea* culture SVU:23 (OR594161).

### 2.6. In Vitro Antifungal Activity

**Figure 5:**
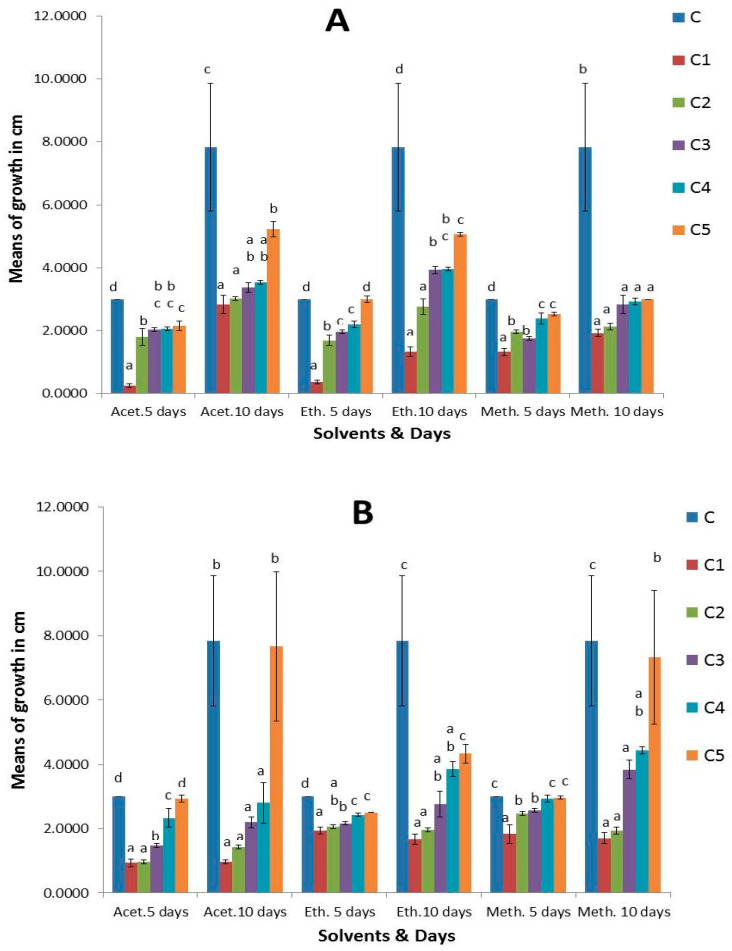
Antifungal activity of (**A**) *S. cinereum* and (**B**) *P. boergesenii* with three organic solvent (acetone, ethyl acetate, and methanol) extracts against *A. niger* at different concentrations (C: control, C1: 100 mg/mL, C2: 80 mg/mL, C3: 60 mg/mL, C4: 40 mg/mL, and C5: 20 mg/mL). Values are means of three replicates ± standard deviation. Statistical significance of differences compared to control: different letters indicate significant differences between treatments at *p* < 0.05 according to the Duncan test.

**Figure 6:**
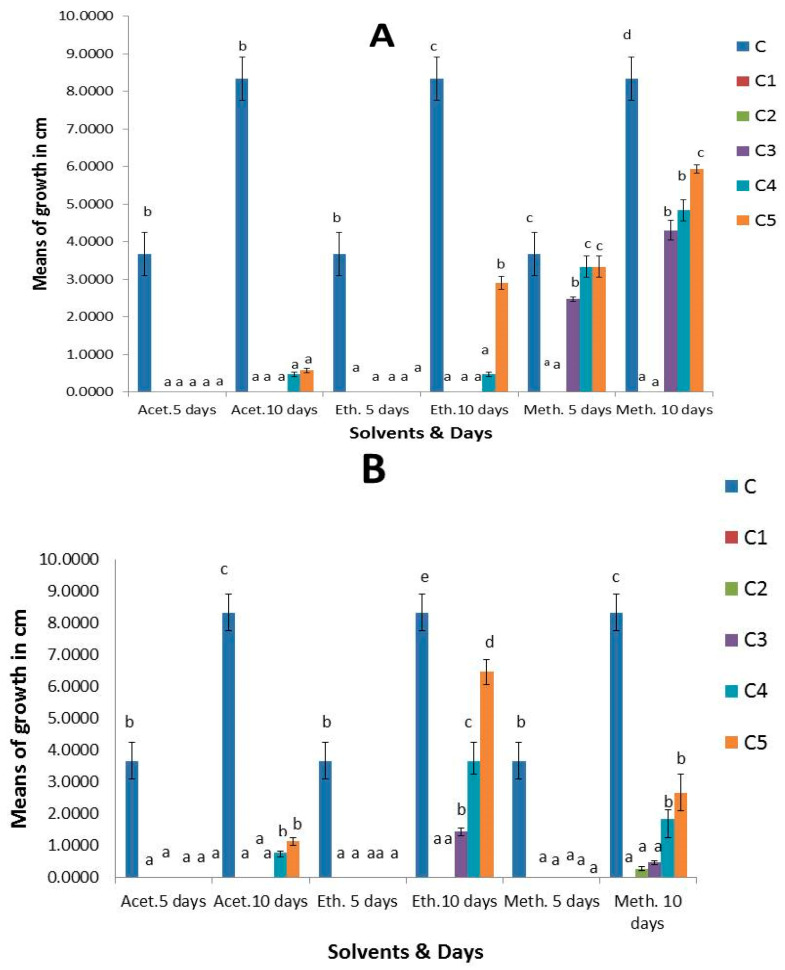
Antifungal activity of (**A**) *S. cinereum* and (**B**) *P. boergesenii* with three organic solvent (acetone, ethyl acetate, and methanol) extracts against *B. cinerea* at different concentrations (C: control, C1: 100 mg/mL, C2: 80 mg/mL, C3: 60 mg/mL, C4: 40 mg/mL, and C5: 20 mg/mL). Values are means of three replicates ± standard deviation. Statistical significance of differences compared to control: different letters indicate significant differences between treatments at *p* < 0.05 according to the Duncan test.

**Figure 7:**
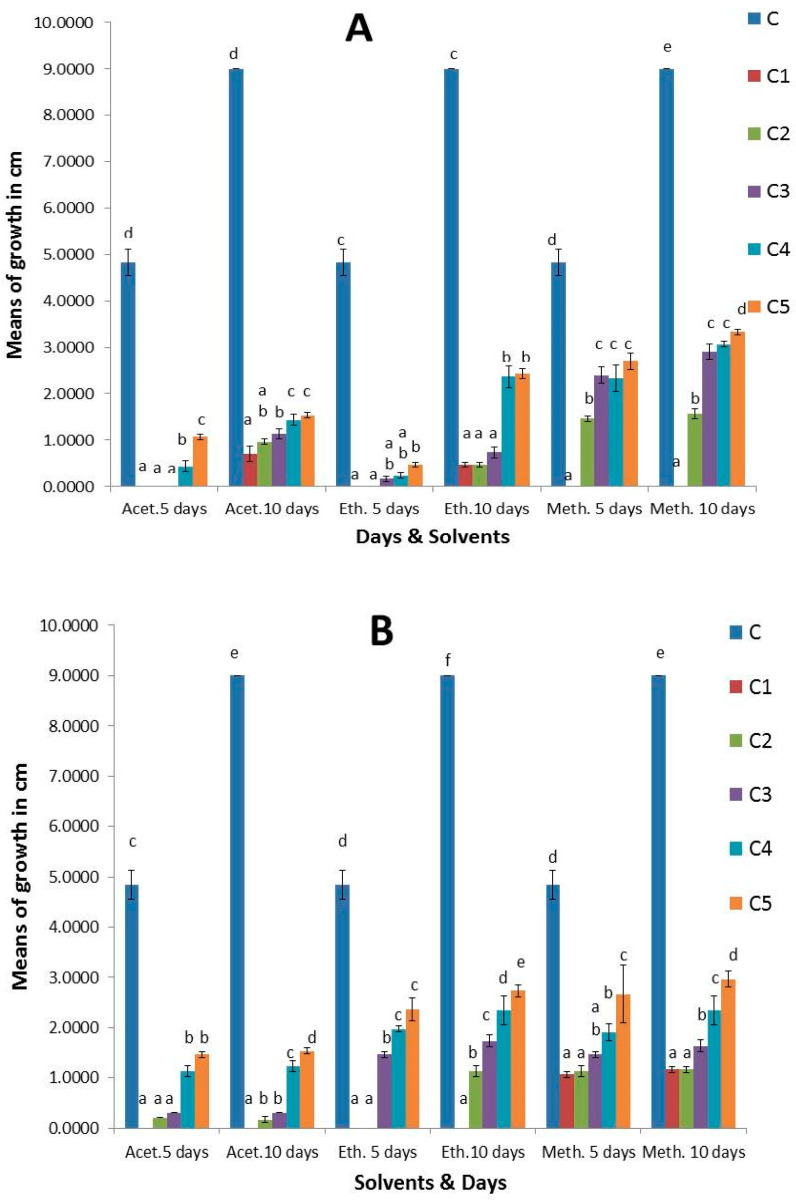
Antifungal activity of (**A**) *S. cinereum* and (**B**) *P. boergesenii* with three organic solvent (acetone, ethyl acetate, and methanol) extracts against *M. irregularis* at different concentrations (C: control, C1: 100 mg/mL, C2: 80 mg/mL, C3: 60 mg/mL, C4: 40 mg/mL, and C5: 20 mg/mL). Values are means of three replicates ± standard deviation. Statistical significance of differences compared to control: different letters indicate significant differences between treatments at *p* < 0.05 according to the Duncan test.

**Figure 8:**
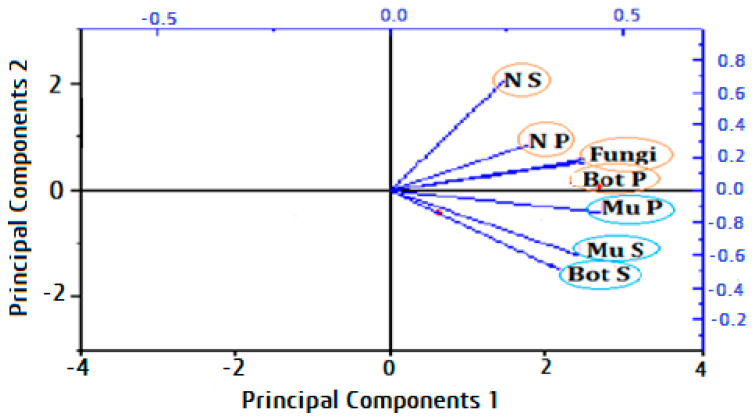
Biplot of principal component analysis manipulating the different patterns of the antifungal activity and their correlations to the tested fungi (*A. niger* with *S. cinereum* = N s, *A. niger* with *P. boergesenii* = NP, *B. cinerea* with *P. boergesenii* = BP, *B. cinerea* with *S. cinereum* = BS, *M. irregularis* with *P. boergesenii* = Mu P, and *M. irregularis* with *S. cinereum* = Mu S).

The results revealed that *S. cinereum*, and *P. boergesenii* extracts with three organic solvents (acetone, ethyl acetate, and methanol) demonstrated varying antifungal activity against the most common species *Aspergillus niger*, *Botrytis cinerea*, and *Mucor irregularis* recovered from strawberry fruits at different concentrations; each effect was significant for a total of 5 and 10 days of incubation. As seen in (Figure S2), the fungal control treated without extracts had the largest colony diameter, which grew with an extended incubation period. After 10 days, it measured between 3 and 9 cm. After the 10-day incubation period, the *S. cinereum* and *P. boergesenii* extracts with three organic solvents (acetone, ethyl acetate, and methanol) at (C1 100 mg/mL, C2 80 mg/mL, C3 60 mg/mL, C4 40 mg/mL, and C5 20 mg/mL) showed strong antifungal activity. Following an incubation period of 10 days, the colony diameters of *Aspergillus niger* treated with *P. boergesenii* extracts at (C1 100 mg/mL) were 1.6 cm for ethyl acetate, 1.7 cm for methanol, and 0.9 cm for acetone. However, after a 10-day incubation period, the colony diameters of *A. niger* treated with *S. cinereum* extracts at (C1 100 mg/mL) were 1.9 cm in methanol, 1.3 cm in acetone, and 2.8 cm in ethyl acetate (Figure 5). For *Botrytis cinerea*, no growth was detected at (C1 100 mg/mL) with *S. cinereum* and *P. boergesenii* with three organic solvent (acetone, ethyl acetate, and methanol) extracts after 10 days of incubation, so they demonstrated a highly significant effect (Figure 6). No growth of *Mucor irregularis* was detected with *P. boergesenii* with acetone and ethyl acetate extracts and with the methanol extract of *S. cinereum* at (C1 100 mg/mL) (Figure 7). Each of the three tested strains was significantly inhibited by the extracts after the 10-day incubation period at (C1 100 mg/mL). In general, no growth of *B. cinerea* and *M. irregularis* was detected with *P. boergesenii* acetone and ethyl acetate extracts. *A. niger* had the lowest mean diameter with *P. boergesenii* acetone extract as illustrated in the biplot of principal component analysis (PCA), which revealed a strong positive correlation between *B. cinerea* and *M. irregularis* that showed no growth with treatment with *S. cinereum* and *P. boergesenii* extracts (Figure 8). However, the two extracts illustrated the lowest inhibition in *A. niger* growth.

Using acetone, ethyl acetate, and methanol as organic solvents of *S. cinereum* and *P. boergesenii* extracts, the study determined the inhibitory percentages for each of the three investigated fungi at (C1 100 mg/mL, C2 80 mg/mL, C3 60 mg/mL, C4 40 mg/mL, and C5 20 mg/mL) after 10 days of incubation. The inhibition percentage of *S. cinereum* and *P. boergesenii* with *B. cinerea* ranged between 28.9–100% and 67–100% for methanol extract, 65–100% and 22–100% for ethyl acetate extract, and 92–100% and 86.3–100% for acetone extracts, respectively. For *M. irregularis,* the inhibition percentage of *S. cinereum* and *P. boergesenii* ranged between 63.3–100% and 67.11–87.11% for methanol extract, 73–94.8% and 69.6–100% for ethyl acetate extract, and 83–92.22% and 83–100% for acetone, respectively. The inhibition percentages of *S. cinereum* and *P. boergesenii* for *A. niger* were 61.6–75.3% and 78.2–3.17% for methanol extract, 35.3–83% and 44.7–78.8% for ethyl acetate extract, and 33.2–63.8% and 2.1–87.7% for acetone, respectively. The acetone extract of *P. boergesenii* was shown to have the strongest in vitro antifungal activity (Table 5 and Figure S3).

### 2.7. Fungal Pathogenicity and Aggressiveness on Strawberry Fruits (In Vivo Antifungal Activity)

The impact of *S. cinereum* and *P. boergesenii* extracts with three organic solvents (methanol, ethyl acetate, and acetone) on the pathogenicity and aggressiveness (in vivo antifungal activity) of *A. niger*, *B. cinerea*, and *M. irregularis* on strawberry fruits after harvesting is shown in Table 6. As could be expected, pathogenicity increased with skin injury. The sort of lesion was different in fruits that were injured and those that were not. The size of the lesion that developed from cutting a strawberry fruit was always equal to the diameter of the fungal colony. A non-wound soft lesion was found outside the colony’s margin in the inoculation state. The aggressiveness value found in both cases matched the lesion as a whole. All extracts were able to reduce the size of the *A. niger*, *B. cinerea*, and *M. irregularis* lesions on strawberry fruits, which was used to measure aggressiveness, as well as the number of infected fruits when compared to the control. In the solvent control by using, acetone, ethyl acetate, and methanol the fungal pathogenicity and aggressiveness were unaffected. *S. cinereum* methanol extract was the most effective against *B. cinerea* on both wounded (30%) and unwounded (10%) fruits. *S. cinereum* acetone and methanol extracts were most effective with *M. irregularis* on both wounded (50%) and unwounded (10%) fruits. *S. cinereum* methanol extract was the most effective on the aggressiveness (1 and 0.5 cm) with *M. irregularis*. Also, for *A. niger S. cinereum,* the methanol extract was the most effective in the case of unwounded fruits (50%). In general, for *S. cinereum*, the methanol extract was the most effective against fungal pathogenicity and aggressiveness. The *P. boergesenii* methanol extract had a great effect (10–50%) against *B. cinerea* and *M. irregularis* on both wounded and unwounded fruits. *P. boergesenii* acetone extract had the greatest impact on *A. niger*. In general, the *P. boergesenii* methanol extracts were the most effective against fungal pathogenicity and aggressiveness.

### 2.8. Extraction and Detection of Botrydial and Dihydrobotrydial Toxins, and Ochratoxin A

**Figure 9:**
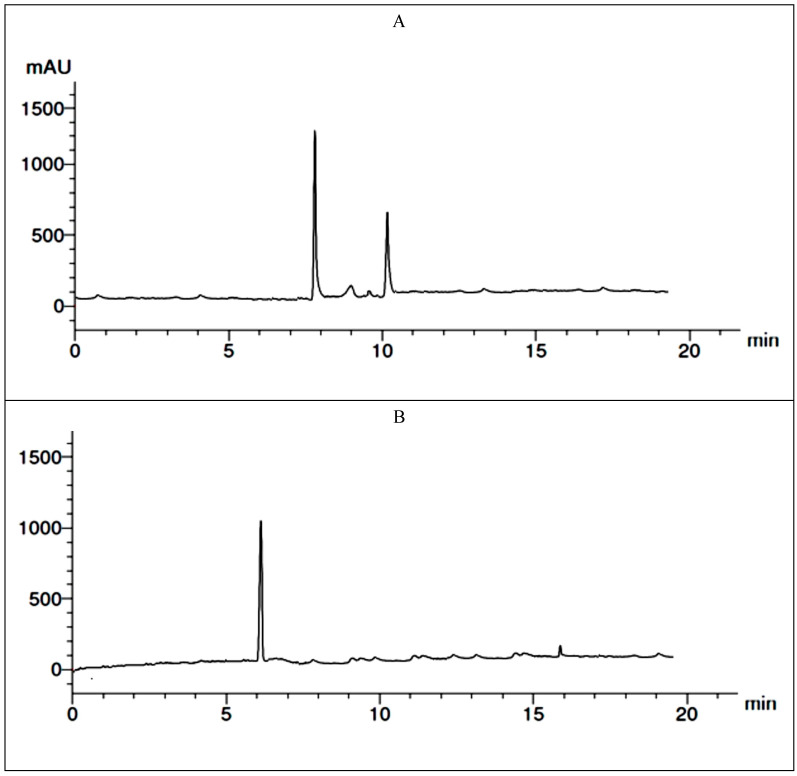
(**A**) HPLC analysis of Botrytis cinerea extract for detection of botrydial and dihydrobotrydial toxins, and (**B**) HPLC analysis of Aspergillus niger extract for detection of ochratoxin A.

Two of the most common fungi were chosen for the detection of mycotoxins. The ability of *B. cinerea* and *A. niger* to produce mycotoxins was investigated (Table 7). *B. cinerea* could produce botrydial toxin with a concentration of 8.14 µg/mL at retention time 11 and dihydrobotrydial toxins with a concentration of 4.26 µg/mL at retention time 11.5. *A. niger* could produce ochratoxin A with a concentration of 10.05 µg/mL at retention time 6 (Figure 9).

## 3. Discussion

Fruit microbial infections can result in significant loss. When treating plant diseases, using commercial fertilizers may have unfavorable side effects. To prevent the infection, another approach must be discovered. Alwaleed [25] shows that seaweed is abundant in novel physiologically active metabolites. Potential bioactive substances include cell extracts and the active components of certain macroalgae [26]. Seaweeds’ ability to fight microbes is influenced by various variables, including growth stages, habitat, the time of year they are collected, and experimental techniques. The research has shown that the effectiveness of antimicrobial agents is dependent on the type of algae species and extraction solvent employed [27].

On average, acetone, ethyl acetate, and methanol are among the organic solvents used to evaluate algal extracts’ antibacterial activity [28]. Acetone is an organic solvent that consistently extracts compounds with antibacterial action with a relatively high degree of efficiency. All three of the solvent extracts were shown to be successful in this investigation in suppressing the pathogens that were tested. All effects considered; acetone proved to be the most successful solvent when it was used to extract seaweed’s antifungal components. Acetone may have a greater dielectric constant than other solvents, which could be the cause. According to the results of the current screening, the acetone extract had the greatest antifungal activity, while methanol and ethyl acetate showed the least, which was consistent with the findings of Padmakumar and Ayyakannu [29].

All of the algae we used for this investigation had antifungal effects and belonged to the *Phaeophyte* category. A straightforward chemical process for finding naturally occurring functional chemicals was described. An approach was devised that encompassed the utilization of uncharted natural resources (seaweeds), ecologically sound extraction methods, and cutting-edge analytical instruments. FTIR and GC–MS chromatography revealed a variety of chemicals found in the *Sargassum cinereum* and *Padina boergesenii* extracts. Numerous chemicals may be responsible for these extracts’ antimicrobial action. Certain antimicrobial activity has been attributed to these compounds, which is consistent with research conducted by Alagic and Das et al. [30,31]. *Padina pavonia* has a high concentration of terpenes and sterols, with 3-furoic acid and phytol serving as the primary terpene components. The presence of alkaloids, phenolics, flavonoids, and tannins is reported in *S. cinereum* and *P. boergesenii*. Alkaloids are reported to be biologically and therapeutically active (e.g., morphine, atropine, and quinine) and have numerous medical applications [31,32,33].

Terpenoids have been suggested to be helpful in the treatment and prevention of several illnesses, including cancer. According to [33], terpenoids are also known to have antibacterial, antifungal, anti-parasitic, antiviral, anti-hyperglycemic, anti-inflammatory, and immunomodulatory qualities. According to [34], flavonoids have antibacterial, free radical scavenger, and antioxidant qualities. In this investigation, the acetone extracts of both algae showed a low level of flavonoid content. Tannins are mild antiseptics to cure diarrhea and stop minor bleeding [35]. In the current study, tannins were found to be poor in ethyl acetic extract and significant in acetonic and methanolic extract. The antioxidant activity of the extracts was proportional to the polarity of the extracting solvents. The extracts prepared using polar solvents, such as methanol and acetone, present the highest efficacy, followed by those prepared using moderately polar solvents like ethyl acetate. According to Matanjun et al. [36], those results can be explained by the fact that the antioxidant capacities are directly related to the secondary metabolites present in each extract and fraction and depend on the antioxidant substances and their nature, quantity, structure, and any molecular interactions that may act synergistically to enhance this activity.

The different phytoconstituents differed depending on the kind of seaweed, its chemistry, the solvents employed, the environment, the time of collection, and the physicochemical characteristics of the water [37]. It was noted that the amount of bioactive chemicals in algae varied depending on the type of algae, the locations and surrounding circumstances [38]. A qualitative phytochemical analysis of algal ethanolic and methanolic extracts identified several beneficial compounds. *Sargassum* has a variety of species that exhibit advantageous biological activity, including anti-inflammatory, antiviral, antibacterial, and anticancer effects [39]. The most abundant six constituents were heptadecane, methyl tetradecanoate, hexadecanoic acid, methyl ester, 11-eicosenoic acid, methyl ester, lucenin, and docosanoic acid, and methyl ester (CAS). Sahar, and Aida [40] reported that seaweed contains a high carotenoid, β-Carotene, and carbohydrates content. Moreover, brown seaweed exhibited good antioxidant activity. According to Djapic’s [41] identification and quantification of the chemical components of seaweeds, algae contain aromatic compounds, alkenes, acyclic alkanes, triterpenes, monoterpenes, and organo-sulfur compounds.

Fruit damage can result from handling, uncontrollable harvesting methods, storage, and transit circumstances, particularly when the production and market areas are far apart [42]. Early data state that transportation from the field to the market accounts for 65% of strawberry losses [43]. In our study, the most common fungal genera isolated from strawberry fruits were *Aspergillus niger*, *Botrytis cinerea*, and *Mucor irregularis* and this was verified in previous reports [5,6,7,8]. *B. cinerea* had the highest total count (152) with a percentage of 38.29 of total fungi and the number of cases of isolation (NCI, out of 30) was 23. According to Barakat, and Al-Masri [44], *B. cinerea* is a prevalent fungal pathogen that causes strawberry infections and harmful grey mold disease. When *B. cinerea* infects several strawberry organs in the field, including fruits during storage, the outcome is poor yield quality [1,45]. The *B. cinerea* infection of strawberries causes a large amount of economic crop loss in both pre-harvest and post-harvest stages, including handling, transportation, and storage. This information is based on reference [46]. According to reports, the establishment of *B. cinerea* grey mold may lead to a 50% decrease in strawberry yield in favorable environmental conditions [47].

It has long been recognized that an extract’s potency is dependent on the examined fungus species, its concentration, and the type of solvent utilized [48,49,50]. Based on each fungus’s mean colony diameter (cm) at five and ten days of incubation, the in vitro antifungal activities of all solvent extracts at various concentrations (20, 40, 60, 80, and 100 mg/mL) against the three common fungi were calculated. These results showed a highly significant effect. From our results, we can conclude that *P. boergesenii* acetone extract had the highest in vitro antifungal activity with the three tested fungi after 10 days of incubation, in agreement with [18,51,52,53,54]. According to Pourakbar et al. [55], at increasing concentrations of macroalgae *Gracilariopsis persica* extract, there was a decrease in the mycelial growth of *Aspergillus niger*, *Penicillium expansum*, *Pyricularia oryzae*, and *Botrytis cinerea*. For all four fungi, 100% inhibition was observed at 800 and 1000 µL.

In general terms, the kind of algal species, the solvent utilized, and the resistance properties of the studied fungus species, all affected how effective the seaweed extracts were as antifungals. The kind of extraction solvent and the kind of algae were the main determinants of the antimicrobial activity of bioactive compounds from marine algae against pathogenic fungi, according to a previous study [56,57]. Support for it came from [58], which observed that, depending on the solvent polarity and the chemical structure of the extracted bioactive material, multiple solvent extracts from the same seaweed could be identified by distinct antimicrobial properties. In the current study, we examined the effects of *S. cinereum* and *P. boergesenii* extracts on the pathogenicity and aggressiveness (in vivo antifungal activity) of *A. niger*, *B. cinerea*, and *M. irregularis* on strawberry fruits after harvesting using three organic solvents: acetone, ethyl acetate, and methanol. The assessment of aggressiveness revealed that all extracts were able to reduce the size of the three evaluated fungal lesions on post-harvest strawberry fruits as well as the proportion of infected fruits as compared to the control. Acetone, ethyl acetate, and methanol did not affect fungal pathogenicity in the solvent control.

There are fewer tests involving in vivo experiments in the literature that concern the evaluation of the antimicrobial activity of crude extracts or fractions of algae [59]. As such, we can consider that the inhibitory action (in vivo and in vitro) of extracts of *Sargassum cinereum* and *Padina boergesenii* with three organic solvents (acetone, ethyl acetate, and methanol) against the three tested fungi on post-harvest strawberry fruits is the first research on this topic in Egypt.

The methanol extracts of both *S. cinereum* and *P. boergesenii* occupied the first position as the most effective extracts against the pathogenicity and aggressiveness of the studied fungi on post-harvest strawberry fruits. The second position was occupied by the acetone extracts of *S. cinereum* and *P. boergesenii*, and these results are in agreement with those of [60,61].

It is currently unclear how antifungal substances that are isolated from macroalgae work. Generally, substances that target the cell wall or membrane directly can affect fungi. Antifungal agents that penetrate the cell can interfere with the mitochondrial respiratory chain, which disrupts homeostasis and stability, and disrupt protein synthesis by interacting with nucleic acids [62]. Sterols found in the fungal membrane are linked to another antifungal mechanism that has been proposed [63,64]. Certain algal chemicals can interact with or prevent the formation of sterols [65]. The naturally occurring sterol that was separated from brown algae is reported to have antifungal and fungistatic properties against *F. culmorum* [66].

In conclusion, the comprehensive analysis of the extracts reveals several bioactive compounds that hold promise for enhancing strawberry management, particularly in disease resistance, and plant health. Among these, phenolic compounds (notably flavonoids and tannins) stand out as prime candidates for continued study. These compounds are known for their strong antimicrobial and antioxidant properties, which could be leveraged to combat common strawberry pathogens. Additionally, terpenoids and alkaloids, with their demonstrated insecticidal and repellent properties, offer potential for developing natural antimicrobial solutions, reducing the reliance on synthetic chemicals. Based on these findings, the authors should focus future research on isolating and testing the efficacy of flavonoids, tannins, and terpenoids in strawberry disease management. These compounds not only offer a sustainable alternative to conventional methods but also align with the growing demand for eco-friendly agricultural practices.

The fungus *B. cinerea* is both pathogenic and saprophytic, infecting the weak or dead portions of the plant before moving on to the remaining healthy plant tissue [67]. The botrydial and its metabolite, which have been found in fungal culture medium, are among the unique fungal metabolites that *B. cinerea* creates based on the botryane skeleton. Mostly produced during plant infection, botrydial is a potent phytotoxin that can cause cell collapse and chlorosis, according to Durán-Patrón et al. [68]. Our results revealed that *B. cinerea* could produce botrydial and dihydrobotrydial toxins with concentrations of 8.14 µg/mL and 4.26 µg/mL, respectively. Many reports verified that the grey mold fungus *B. cinerea* produces two major phytotoxins, botrydial and dihydrobotrydial [67,69,70,71,72,73].

Wilhem first identified ochratoxin A (OTA) in 1965 as a metabolite of *Aspergillus ochraceus*. Subsequent research revealed that ochratoxins could be produced by a wide range of fungal species, including *Penicillium* species like *P. expansum*, *P. verrucosum*, and *P. chrysogenum* and *Aspergillus* species like *A. niger*, *A. melleus*, and *A. albertensis* [74,75]. The International Agency for Research on Cancer classified OTA as a Group 2B carcinogen due to its nephrotoxic, immunosuppressive, teratogenic, and carcinogenic characteristics [76,77]. *Aspergillus niger* is a widespread natural food and feed contamination fungus, and it is also one of the most prominent industrial filamentous fungal species used in biotechnology [78,79]. In 1987, the Food and Drug Administration (FDA) stated that, according to many studies, strains of *A. niger* isolated from a variety of substrates, including Colombian coffee beans, raisins, grapes, maize, mixed feeds, and component raw materials, could produce OTA [80,81].

In our investigation, *A. niger* could produce OTA with a concentration of 10.05 µg/mL. Similar to this, earlier studies showed that, depending on the food source and the culture conditions, between 1 and 41% of *A. niger* strains isolated from foods like dried vine fruits and wine grapes were able to produce OTA [82,83,84,85,86,87,88].

According to previous literature, *Mucor irregularis* could cause mucormycosis in immunocompromised patients but it could not produce specific mycotoxins [89,90,91,92].

## 4. Materials and Methods

### 4.1. Seaweed Sample Collection

In September 2022, two brown seaweed species, *Sargassum cinereum* and *Padina boergesenii*, were collected from Hurghada on Egypt’s Red Sea coast. The gathered algae were identified according to [93]. After being carefully cleaned with seawater to remove any epiphytes and sand particles, the samples were brought to the lab in sterile containers and then cleaned with tap and distilled water. Spread out on plates, the algae samples were ground using an electric blender after being dried in the shade [20].

### 4.2. Preparing Seaweed Extracts

0.5 kg of algae powder was submerged separately in 1 L of acetone, ethyl acetate, and methanol in a rotary shaker (120 rpm) at room temperature for seven days. The mixture was filtered via Whatman No. 1 sterile filter paper using a rotary evaporator to remove the solvent under reduced pressure [25].

### 4.3. Qualitative Phytochemical Analysis

Each solvent extract was subjected to primary phytochemical analysis using conventional qualitative techniques to ascertain the presence of alkaloids, terpenoids, flavonoids, tannins, polyphenols, and quinones. Once the residue, or crude extracts, were suspended in dimethyl sulfoxide (DMSO), 1 g/mL, to a final concentration, they were stored in the refrigerator for subsequent use [94]. Five concentrations of the algal extracts (20, 40, 60, 80, and 100 mg/mL) were evaluated.

### 4.4. FTIR Spectroscopy

FTIR spectroscopy (Perkin Elmer Spectrum 2) (USA PerkinElmer Inc., Atlanta, GA, USA) was used to identify the existence of distinctive peaks and their functional groups in various crude extracts of *S. cinereum* and *P. boergesenii*. The wavelength range for FTIR spectra was between 450 and 4000 cm^–1^. Three separate analyses were performed, and each analysis verified the spectrum for every extract [40].

### 4.5. GC–MS Analysis

GC–MS was used to evaluate seaweed extracts. For GC–MS, the material was injected into an HP-5 column (30 m × 0.25 μM film thickness). Gas was transported using the PerkinElmer Clarus 580/560 S model system (USA PerkinElmer Inc.) at a flow rate of 0.8 mL/min. The GC oven’s temperature was set to rise from 60 to 250 °C at a pace of 2 °C per minute. Relative area measurements (as a proportion of the total volatile content) were directly determined from the total ion current. By comparing the mass spectra of the crude extracts of *S. cinereum* and *P. boergesenii* with those of reference compounds kept in the NIST library, the identification of the phytochemicals that were previously unknown was verified [32,40].

### 4.6. Gathering Samples of Strawberries

Thirty samples of contaminated (Fragaria x ananassa) were randomly selected in the winter of 2022 from different marketplaces in Qena, Upper Egypt (26°10,000″ N 32°43,000″ E). The samples were brought to a microbiology facility for fungal isolation in sterilized polyethylene bags (Supplementary Figure S1) [11].

### 4.7. Mycobiota Analysis

The media used for the isolation of fungi was potato dextrose agar (PDA) medium, which consisted of 200.0 g of potato, 20.0 g of dextrose, and 15.0 g of agar (Merck, Darmstadt, Germany). The bacteriostatic agent employed in the medium was chloramphenicol (0.05 g/L). According to Pitt, and Hocking [95], infected strawberry fruits were utilized to isolate fungus using a baiting technique (Figure S1). Before being sliced into equal segments (approximately 1 cm each), strawberry fruits were disinfected for one minute with 1% sodium hypochlorite, washed three times with sterilized distilled water, and dried with filter papers. Four pieces of sliced fruit were put on PDA-medium-coated plates. For every sample, three plates were made. The cultures had been kept for seven days at 28 °C. The developing fungal species were examined using a light microscope with a 40 magnification, and three plates were made for each sample. The cultures were kept at 28 °C for seven days. The growing fungal species were examined and morphologically characterized using a light microscope (40 magnification) based on macro- and microscopic characteristics. Fungal species were identified morphologically by Domsch et al. [96].

### 4.8. DNA Extraction

By the directions included with the QIAamp DNeasy Plant Mini kit, fungal cultures were cultivated on the PDA at 25 °C for seven days before being used to extract DNA. For further processing, 100 mg of fungal mycelia were frozen for 24 h at 80 °C [97].

### 4.9. Polymerase Chain Reaction, Amplification of 5.8S rDNA, and Gene Sequencing

The amplification of 5.8S rDNA was made using the universal primer pair ITS1 (50TCCGTAGGTGAACCTGCGG03) and ITS4 (50TCCTCCGCTTATTGATATGC03) and gene sequencing was made according to [98]. The nucleotide sequences procured by the local BLAST (http://blast.ncbi.nlm.nih.gov/Blast.cgi, accessed on 10 September 2023) were tested and emulated with identical sequences in the Gen-Bank.

### 4.10. In Vitro Antifungal Activity

Fungal spores were grown and incubated at 28 °C for seven days in Petri dishes that were filled with (PDA). The concentrations of C1: 100 mg/mL, C2: 80 mg/mL, C3: 60 mg/mL, C4: 40 mg/mL, and C5: 20 mg/mL were made by aseptically adding one milliliter of each of the tested extracts to sterile PDA medium that had been melted. The control plates (C) filled with (PDA) only were prepared. This was done in triplicate for each. Upon cooling down, the agar surface was inoculated with fungal inoculums. A 10-day incubation period at 28 °C was conducted for each Petri dish. After five and ten days of incubation, the diameter of the colony was measured along with the radial growth of the fungal mycelium. The growth inhibition measure for antifungal activity was the mean colony diameter (cm) ± SD. Once the growth of mycelia in the control plate reached the Petri plate’s outer border after ten days of incubation, the percentages of each fungal strain that was inhibited were determined. The % of inhibition was computed using the following formula:

Inhibition% = R − r/R × 100

where the radial growth of fungal mycelia on the control plate is represented by (R), and the radial growth of fungal mycelia on the plate treated with *Sargassum cinereum* and *Padina boergesenii* extracted with acetone, ethyl acetate, and methanol are represented by (r) [99].

### 4.11. Fungal Pathogenicity and Aggressiveness (In Vivo Antifungal Activity) on Strawberry Fruit

The pathogenicity and aggressiveness of the most common fungal species isolated from strawberry fruits were studied in vivo to see if the extracts *Sargassum cinereum* and *Padina boergesenii*, using three organic solvents (acetone, ethyl acetate, and methanol), had an inhibitory action. For the experiment, we used healthy strawberry fruits that were uniform in size and shape, according to El-Shahir et al. [99]. Following a quick rinse under running water, the fruits were submerged for two minutes in a 1% sodium hypochlorite solution. They were dried in a laminar flow hood after being cleansed with sterile distilled water. Each strawberry fruit had two parts cut and was marked. One piece, measuring 2 mm deep and 3 mm wide, was wounded using a sterile scalpel, while the other portion remained intact. Three organic solvents extracts (acetone, ethyl acetate, and methanol) were used individually, to spray 10 mL of 100 mg/mL *Sargassum cinereum* and *Padina boergesenii* over each 200 g of fruit. The fruit was then allowed to dry for 30 min in a laminar flow cabinet. The sprayer was held 30 cm away from the fruit, creating a fine mist, to ensure uniform dispersion. Conidia of the fungi were removed separately by flooding each petri dish (using 7-day-old PDA culture media) with 10 mL of sterile distilled water and scrubbing the surface with a glass rod. The suspension was completed to 100 mL (1 × 10^7^ spores /mL) to be used for fungal spray inoculation. Each strawberry was sprayed using a 2 mL spore suspension of the most frequent fungus species identified from strawberry fruits, both in the healthy and wounded portions. Ten replicates of each treatment were made. As positive controls, individual fruits infected with the fungus species were used. To provide a third kind of control (solvent control), infected fruits were sprayed with organic solvents, removing any possibility that the organic solvents would have an inhibitory effect. The fruits were incubated at 25 °C for five days. After incubation, the number of infected fruits was recorded, and the lesion’s extent was evaluated. Aggression was assessed by measuring the total extent of the lesion, and pathogenicity was determined by counting the number of infected fruits.

### 4.12. Extraction and Detection of Botrydial and Dihydrobotrydial Toxins

A modification of the procedure of Liñeiro et al. [100] was used to construct the isolation of fungal metabolites. After being grown on PDA culture for three days, *B. cinerea* was transferred to individual 500 mL reagent bottles (10 bottles total, with 1 cm of agar plugs per bottle). Each bottle had approximately 250 mL of modified Czapek-Dox medium, which was constantly shaken at 250 rpm for 13 days at a constant room temperature of 22 °C with 12 h light/dark cycles. After gathering all of the liquid broths, ethyl acetate was used to extract the contents (3 × 0.5 vol.). To remove the residue, the combined organic extract was dried over anhydrous Na_2_SO_4_ and concentrated at a lower pressure.

### 4.13. Extraction and Detection of Ochratoxin A

The extraction and detection of ochratoxin A was done according to Samson et al., and Ben Miri et al. [101,102]. Each 500 mL sterile Erlenmeyer flask was filled with 300 mL of the liquid yeast extract sucrose (YES) medium, which contains 40 gm of sucrose and 20 gm of yeast extract per liter (3 duplicates). Following sterilization, three agar discs derived from cultures on YES agar plates that were seven days old were added to each flask. The flasks were then incubated for 15 days at 28 °C. After the incubation period, the cultures were filtered, and each flask was extracted using 100 mL chloroform for a whole day at 20 °C and 160 rpm of shaking. To separate the mixture into an upper layer made up of spores and mycelia and a lower layer made up of chloroform containing extracted toxins, the mixture was then placed onto a separator funnel. Subsequently, the lower layer (the final extract of chloroform) was transferred to a clean dry flask and was evaporated in a water bath (50 °C). Then, dry powder remained in a flask. The residue was diluted with chloroform to 1 mL.

### 4.14. Chromatographic Analysis

The Hewlett-Packard HP1050 liquid chromatograph (Hewlett-Packard, Palo Alto, CA, USA) is the main component of the HPLC system. It is equipped with a Rheodyne sample valve that has a 20 mL loop installed and an HP diode array detector (model 1050, Phoenix, with Macro Spectro software version 2.0). Spherisorb ODS-2, 5 mm, 250 mm was the analytical column (Phase Separations, Deeside, Chwyd, UK). Before being placed into the chromatograph, the sample and standard solutions were subjected to a 30 s sonication. The mobile phase consisted of methanol/water (80:20) with 300 mg ZnSO_4_·H_2_O/L and a flow rate of 0.7 mL/min. For insertion chromatograms, 250 nm was the wavelength. The toxin standards were used to create a calibration curve that correlated peak area with concentration to achieve the quantification objective. The peak identity was established by contrasting the spectrum of the standard with the presumptive positive peak in the sample after normalization [103].

### 4.15. Statistical Analysis

Data were statistically analyzed using the SPSS program. An analysis of variance (ANOVA) was carried out using a general one-way model (*p* < 0.05).

## Figures and Tables

**Table 1:** Phytochemicals of *S. cinereum* extracted with different solvents.

Phytochemicals	Different Solvents		
	Ethyl Acetate (Poor-Polar)	Acetone (Middle-Polar)	Methanol (Polar)
Alkaloids	+++	-	+++
Terpenoids	+	+	+
Flavonoids	+	-	+
Tannins	-	+	-
Polyphenols	+++	++	+
Quinones	++	+++	+++

+, Low presence; ++, Moderate presence; +++, High presence; -, Absence.

**Table 2:** Phytochemicals of *P. boergesenii* extracted with different solvents.

Phytochemicals	Different Solvents		
	Ethyl Acetate (Poor-Polar)	Acetone (Middle-Polar)	Methanol (Polar)
Alkaloids	-	-	+
Terpenoids	++	+	-
Flavonoids	+	-	+
Tannins	-	+	+
Polyphenols	+++	++	+
Quinones	+	+	+++

+, Low presence; ++, Moderate presence; +++, High presence; -, Absence.

**Table 3:** Phytochemical components of various solvent extracts of *S. cinereum* and *P. boergesenii* identified by GC–MS spectroscopy.

Peak No.	Compound Name	Molecular Weight	Chemical Structure and Molecular Formula	Retention Time	Biological Activity According to [25,26]
1	Heptadecane	240	C_17_H_36_	17.68	Exhibited antibacterial and antifungal activity
2	Methyl tetradecanoate	272	C_15_H_30_O_2_	18.17	Antioxidant and antibacterial
3	2-Hexadecen-1-ol, 3,7,11,15-tetramethyl-, [R-[R*,R*-(E)]]-(CAS)	296	C_20_H_40_O_2_	19.64	
4	Phytol, acetate	338	C_22_H_42_O_2_	19.73	Antibacterial activity
5	7,10-Hexadecadienoic acid, methyl ester	266	C_17_H_30_O_2_	20.25	
6	9-Hexadecenoic acid, methyl ester, (Z)-	268	C_17_H_32_O_2_	20.68	
7	Hexadecanoic acid, methyl ester	270	C_17_H_34_O_2_	21.09	Antioxidant
8	Phytol	296	C_20_H_40_O	22.60	
9	9,12,15-Octadecatrienoic acid, methyl ester, (Z,Z,Z)-	292	C_19_H_32_O_2_	23.22	Antibacterial, anticandidal, and anticancer
10	Heptadecanoic acid, 16-methyl-, methyl ester	298	C_19_H_38_O_2_	23.46	
11	11-Eicosenoic acid, methyl ester	324	C_21_H_40_O_2_	25.49	Alpha-glucosidase inhibitor activity, detergents, and lubricants
12	Lucenin	610	C_27_H_30_O_16_	27.67	
13	Docosanoic acid, methyl ester (CAS)	354	C_23_H_46_O_2_	29.95	

**Table 4:** Total counts (calculated per three replicates × 4 pieces), the number of cases of isolation (NCI) out of 30 samples, and the occurrence remarks (OR) of fungal genera and species isolated from strawberry fruits.

Fungi	TC	%	NCI and OR
*Acremonium strictum*	10	2.52	4 L
*Alternaria alternata*	21	5.29	5 L
*Aspergillus*	68	17.13	21 H
*A. flavus*	8	2.02	4 L
*A. fumigatus*	3	0.76	2 R
*A. niger*	57	14.36	19 H
*Botrytis cinerea*	152	38.29	23 H
*Fusarium oxysporum*	6	1.51	4 L
*Humicola grisea*	7	1.76	4 L
*Mucor irregularis*	67	16.88	20 H
*Penicillium chrysogenum*	7	1.76	4 L
*Phoma medicaginis*	59	14.86	7 M
*Rhizopus oryzae*	9	2.27	3 L
Gross total count	397		
Number of genera	10		
Number of species	12		

TC, total count of fungi; OR, occurrence remarks (out of 30 samples); H, high occurrence, from 15 to 30 cases; M, moderate occurrence, from 7 to 14 cases; L, low occurrence, from 3 to 6 cases; and R, rare occurrence, from one and two cases.

**Table 5:** Growth inhibition (%) of *Botrytis cinerea*, *Mucor irregularis,* and *Aspergillus niger* at 10 days of incubation with different solvent extracts of *Sargassum cinereum* and *Padina boergesenii*.

Fungi	Extract Concentration (mg/mL)		*Sargassum cinereum*			*Padina boergesenii*	
		Methanol	Ethyl Acetate	Acetone	Methanol	Ethyl Acetate	Acetone
	100	100.00	100.00	100.00	100.00	100.00	100.00
	80	100.00	100.00	100.00	96.87	100.00	100.00
***Botrytis cinerea***	60	48.19	100.00	100.00	94.46	83.13	100.00
	40	42.17	94.46	94.46	78.31	55.90	90.84
	20	28.92	65.06	93.25	67.95	22.17	86.39
	100	100.00	94.89	92.22	87.11	100.00	100.00
	80	82.67	94.89	89.33	87.11	87.44	98.22
***Mucor*** ***irregularis***	60	67.78	91.89	87.44	81.89	80.78	96.67
	40	66.67	73.78	84.11	74.11	74.11	86.33
	20	63.33	73.00	83.00	67.11	69.67	83.00
	100	75.35	83.01	63.86	78.29	78.80	87.74
	80	72.80	64.75	61.30	75.35	74.97	81.74
***Aspergillus niger***	60	63.86	49.81	53.64	51.09	64.75	71.90
	40	62.58	49.43	54.92	6.39	50.70	64.24
	20	61.69	35.38	33.21	3.17	44.70	2.17

Shades of green indicate high growth inhibition (%) and shades of red indicate low growth inhibition (%) of extracts.

**Table 6:** Effect of different *Sargassum cinereum* and *Padina boergesenii* extracts on the pathogenicity and aggressiveness of *Botrytis cinerea*, *Mucor irregularis,* and *Aspergillus niger* on strawberry fruits.

Treatments	*Botrytis cinerea*				*Mucor irregularis*				*Aspergillus niger*			
	Pathogenicity ^1^		Aggressiveness ^2^		Pathogenicity ^1^		Aggressiveness ^2^		Pathogenicity ^1^		Aggressiveness ^2^	
	W	U	W	U	W	U	W	U	W	U	W	U
Positive control ^3^	100	100	3.4	3.1	100	100	3.8	3.6	100	100	4.2	3.9
Solvent control ^4^/Methanol	100	100	3.4	3.5	100	100	3.8	3.5	100	100	4.1	4
Solvent control ^4^/Ethyl acetate	100	100	3.6	3.7	100	100	3.9	3.6	100	100	4	3.8
Solvent control ^4^/Acetone	100	100	3.8	3.6	100	100	3.7	3.6	100	100	4	3.9
*S. cinereum*/Methanol	30	10	1.5	0.3	50	10	1	0.5	100	50	3	2
*S. cinereum*/Ethyl acetate	70	20	3	2.5	100	30	3.5	3.1	100	70	3.5	3.3
*S. cinereum*/Acetone	40	10	2	0.5	50	10	1.5	0.9	100	70	3.5	3.2
*P. boergesenii*/Methanol	20	10	0.3	0.1	50	15	0.2	0.1	100	60	3.2	3
*P. boergesenii*/Ethyl acetate	50	20	1.2	1	100	20	0.3	0.1	100	55	3.5	3.2
*P. boergesenii*/Acetone	30	15	1.2	0.9	60	20	0.3	0.2	100	50	2.9	2.5

^1^ % of infected fruits. Mean of two independent experiments. ^2^ Mean lesion diameter (cm). Mean of ten replicates from two independent experiments. ^3^ Inoculated fruits untreated with algae extracts or solvents. ^4^ Inoculated fruits sprayed with different solvents.

**Table 7:** HPLC analysis of mycotoxins (calculated by µg/mL of fungal extracts).

Fungi	Mycotoxins	Concentrations
*Botrytis cinerea*	Botrydial	8.14
	Dihydrobotrydial	4.26
*Aspergillus niger*	Ochratoxin A	10.05

## Data Availability

The data presented in this study are available on request from the corresponding author.

## Supplementary Materials

The following supporting information can be downloaded at: https://www.mdpi.com/article/10.3390/plants13223115/s1, Figure S1. Some samples of infected strawberries fruits; Figure S2. Heatmap showing the colony diameter (cm) of different pathogenic fungi treated with different solvent extracts, (M) methanol, (EA) ethyl acetate, and (AC) acetone, of *Sargassum cinereum* and *Padina boergesenii*. (NP) *A. niger* with *P. boergesenii*, (NS) *A. niger* with *S. cinereum*, (MU P) *M. irregularis* with *P. boergesenii*, (MU S) *M. irregularis* with *S. cinereum*, (BOT P) *B. cinerea* with *P. boergesenii*, and (BOT S) *B. cinerea* with *S. cinereum*; Figure S3. Pearson’s correlation analysis showing the correlation coefficients (r) of the different seaweed extracts of *Sargassum cinereum* and *Padina boergesenii* and their antifungal activities against the tested pathogenic fungi. (NP) *A. niger* with *P. boergesenii*, (NS) *A. niger* with *S. cinereum*, (MU P) *M. irregularis* with *P. boergesenii*, (MU S) *M. irregularis* with *S. cinereum*, (BOT P) *B. cinerea* with *P. boergesenii*, and (BOT S) *B. cinerea* with *S. cinereum* using different concentrations of extracts; Figure S4. Standards of (A) botrydial, dihydrobotrydial, and (B) ochratoxin A.
