# Supplementary material for: Antifungal Properties of Sargassum cinereum and Padina boergesenii Extracts Against Fungi Associated with Strawberry Fruits Concerning Mycotoxin Production
Source: Plants (Basel). 2024 Nov 5;13(22):3115. doi: 10.3390/plants13223115 (PMC11597142; doi:10.3390/plants13223115)
Supplement: Supplementary file 1 [file plants-13-03115-s001.zip › plants-3165537-supplementary.pdf]

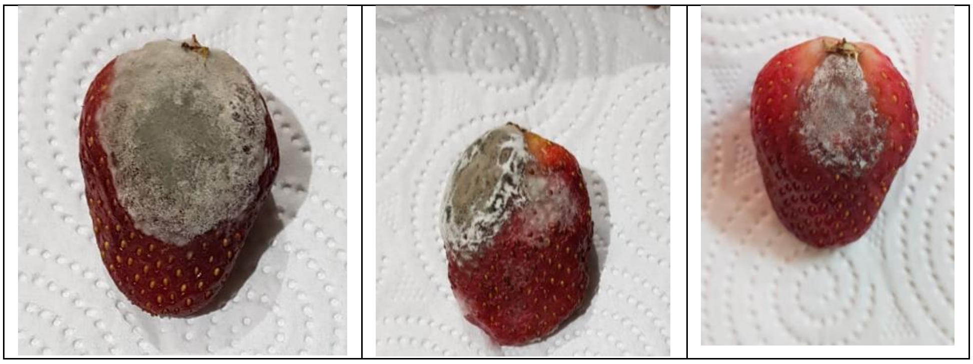

Figure S1: Some samples of infected strawberries fruits.

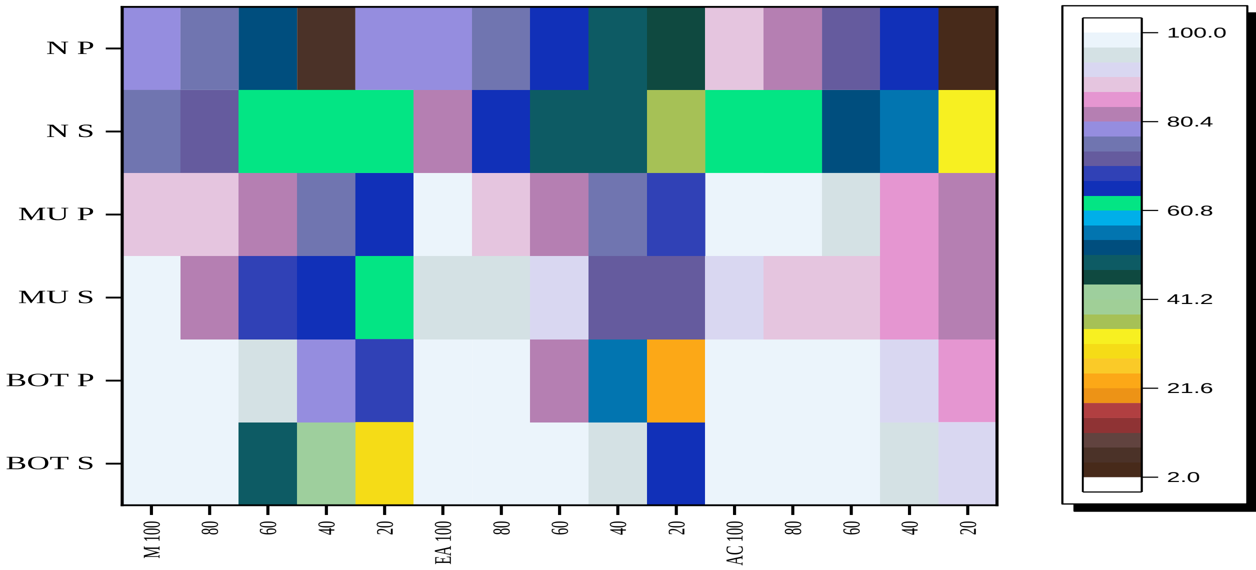

Figure S2. Heatmap showing the colony diameter (cm) of different pathogenic fungi treated with different solvent extracts (M) methanol, (EA) ethyl acetate, and (AC) acetone at different concentration (C1 100 mg/mL, C2 80 mg/mL, C3 60 mg/mL, C4 40 mg/mL, and C5 20 mg/mL) of *Sargassum cinereum* and *Padina boergesenii*. (NP) *A. niger* with *P. boergesenii*, (NS) *A. niger* with *S. cinereum*, (MU P)*M. irregularis* with *P. boergesenii*, (MU S) *M. irregularis* with *S. cinereum*, (BOT P) *B. cinerea* with *P. boergesenii*, and (BOT S) *B. cinerea* with *S. cinereum.*

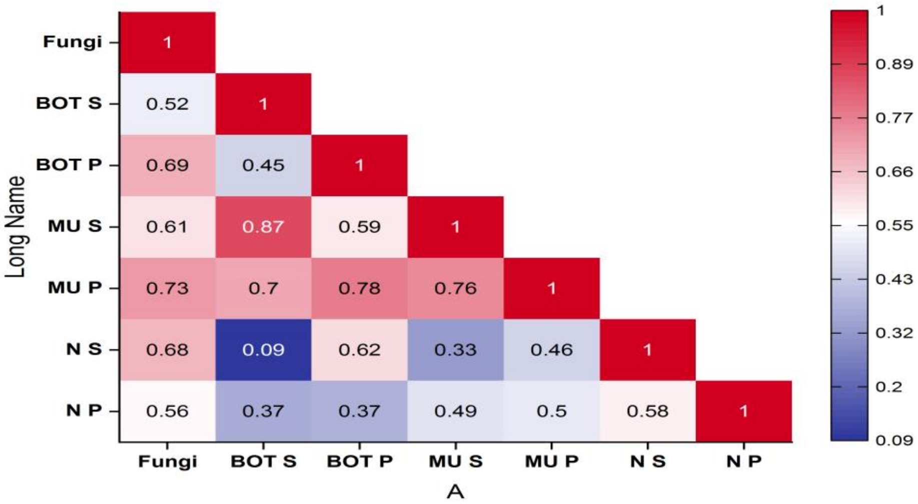

Figure S3

Pearson’s correlation analysis showing the correlation coefficients (r) of the different seaweed extracts of *Sargassum cinereum* and *Padina boergesenii* and their antifungal activities against the tested pathogenic fungi. (NP) *A. niger* with *P. boergesenii*, (NS) *A. niger* with *S. cinereum*, (MU P) *M. irregularis* with *P. boergesenii*, (MU S) *M. irregularis* with *S. cinereum*, (BOT P) *B. cinerea* with *P. boergesenii*, and (BOT S) *B. cinerea* with *S. cinereum* using different concentration of extracts.

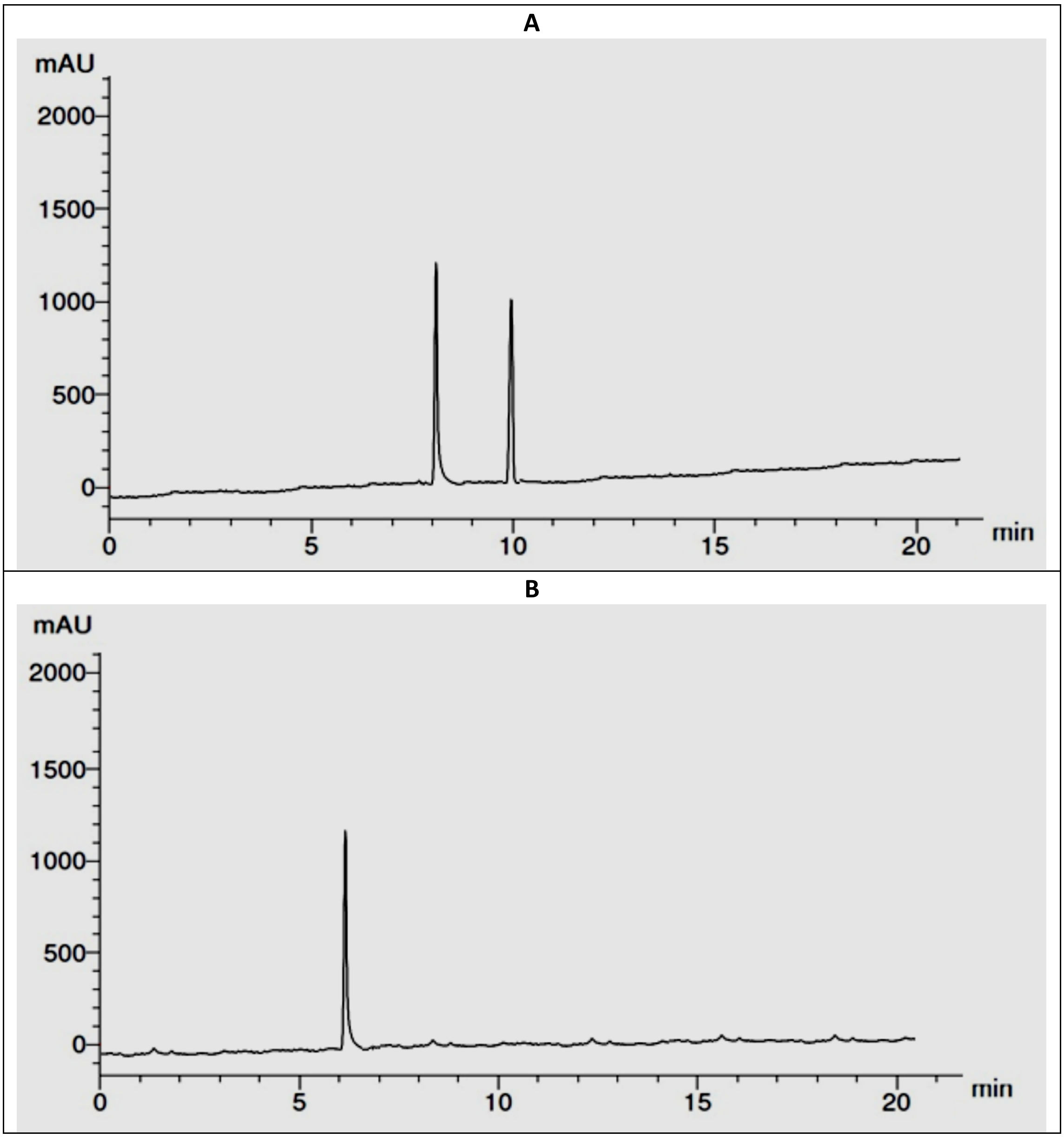

Figure S4: Standards of **(A)** Botrydial, Dihydrobotrydial and **(B)** ochratoxin A.
